# Analytical construction and visualization of nonlinear waves in the (2+1) dimensional Kadomtsev-Petviashvili-Sawada-Kotera-Ramani equation with stability analysis

**DOI:** 10.1038/s41598-025-18737-w

**Published:** 2025-09-15

**Authors:** Mahy Ahmed, Hamdy M. Ahmed, Niveen Badra, Islam Samir

**Affiliations:** 1 Department of Physics and Mathematics Engineering, Faculty of Engineering, Ain Shams University, Cairo, Egypt; 2 Department of Physics and Engineering Mathematics, Higher Institute of Engineering, El Shorouk Academy, Cairo, Egypt

**Keywords:** KPSKR equation, Nonlinear wave propagation, Hyperbolic wave structures, Graphical analysis, Mathematics and computing, Optics and photonics, Physics

## Abstract

In this study, we investigate the (2+1)-dimensional Kadomtsev–Petviashvili–Sawada–Kotera–Ramani (KPSKR) equation, a physically significant model describing nonlinear wave phenomena in higher-dimensional spaces. Utilizing the improved modified extended tanh-function method, we derive a diverse spectrum of exact analytical solutions. These include bright solitons, singular solitons, singular periodic waves, and hyperbolic function solutions. The physical characteristics and dynamical behaviors of the obtained solutions are further elucidated through comprehensive two-dimensional and three-dimensional graphical visualizations, offering insight into the complex wave structures governed by the KPSKR equation. The results highlight the versatility of the proposed method and the rich nonlinear dynamics inherent in the model.

## Introduction

A class of differential equations well known as nonlinear evolution equations (NLEEs) describes the evolution of physical, biological, or other systems over time. These equations are “nonlinear,” meaning that the relationship between the variables can interact in complex ways, leading to behaviors like waves, solitons, turbulence, or pattern formation. Due to the vital importance of (NLEEs) in clarifying nonlinear phenomena and their applications in a variety of domains, including chemistry, quantum mechanics, fluid dynamics, meteorology, and optics, the study of these equations is growing throughout time^1–8^.

In 1970, Kadomtsev and Petviashvili introduced a two-dimensional extension of the well-known KdV equation, which was the Kadomtsev–Petviashvili equation^9^, the KP equation characterized by soliton solutions obtained from the inverse scattering transform^10^. Therefore, it is still a crucial point in the field of integrable equations, especially in (2+1) dimensions. It is used for clarifying many physical properties in various domains. Then the Sawada–Kotera equation was revealed by Sawada and Kotera in 1974^11^. By Guo in 2022, the Kadomtsev–Petviashvili equation was combined with the Sawada–Kotera equation forming Kadomtsev–Petviashvili–Sawada–Kotera–Ramani equation^12^.

Basic studies on the KP hierarchy itself, such as work on the complex characteristics of the first equation of the Kadomtsev–Petviashvili hierarchy^13^, are critical for understanding the mathematical underpinnings and integrability of equations like KPSKR. Such investigations develop the foundation of Lax pairings, conservation laws, and inverse scattering, which is then used to analyze more particular members such as KPSKR. The KPSKR equation in (2+1) dimensions was a higher-dimensional generalization of integrable systems, meaning it has soliton solutions, Lax pairs^12^, and other properties associated with integrable systems, making it a rich subject of study in mathematical physics. It is also characterized by strong nonlinearity, allowing for complex wave interactions in multidimensional settings, which challenge researchers with their intricate analytical and numerical solutions. The KPSKR equation is employing to model complicated nonlinear wave phenomena such as fluid dynamics, nonlinear optics, condensed matter physics and plasma physics.

The KPSKR equation in (2+1) dimensions is expressed as follows^14^:

1 $$\begin{aligned} u _{xt}+(3 u^2+u_{xx})_{xx}+(15 u^3+15 u u_{xx}+u_{xxxx})_{xx}+\sigma u_{yy} =0. \end{aligned}$$

Here, u=u(x,y,t) denotes the amplitude of the wave, representing its variation across spatial coordinates x and y as well as over time t, The coefficient $\sigma$ is an arbitrary constant that determines the specific characteristics of the equation.

As mentioned, the KPSKR equation provides solutions for solitary waves—localized waves that retain their original shape and velocity after interactions. These solutions can be found using methods such as the bilinear Hirota method^15–17^, the inverse scattering transform^18,19^, and the Buckland transform^20–22^.

For example, research on solitons, multibreathing molecules, and hybrid solutions to related two-dimensional (2+1) KdV-Sawada-Kotera-Ramani equations^23^ highlights the current trend. These works apply Hirota’s bilinear formulation to produce complex coherent structures in which solitons remain bound in a particle-like steady state or interact with breathers (localized oscillatory modes). This demonstrates that the emphasis is now on studying complicated multicomponent interactions and solutions instead of isolated waves. Moreover, lump solutions—localized, rogue-wave-like structures that vanish in all spatial directions—have emerged as a key research area. The dynamical analysis of lump solutions to (3+1)D equations and their reductions^24^ and the investigation of anomalous mass scattering phenomena in lumps in extended KP equations^25^ show that these structures have a rich dynamical behavior, including intriguing scattering properties and energy sharing during interactions.This links our purely mathematical attempt to the concrete physical conditions predicted by the basic KP model^13^, highlighting the practical importance of developing new solution methods that help better describe complex wave dynamics in fluids. This is supplemented by research on bright-dark lump wave solutions in high-dimensional BKP equations^26^, which shows the universality and adaptability of these localized solutions across many integrable hierarchies. While the bilinear technique is unrivaled in finding these novel solutions, it often requires a high level of intuition and expertise to develop the right ansatz (for example, the form of the perturbation function). The improved modified extended tanh function approach again comes to the fore as it provides a highly structured and algorithmic framework. During this paper, solutions for solitary waves are obtained by employing the improved modified extended Tanh method^27^ on the KPSKR problem^14^. The novelty of the method lies in deriving new exact results for the KPSKR problem, including singular, periodic solutions and bright soliton solutions consistently and straightforwardly. This technique reduces the challenge of solving the NLEE equation to solving a system of algebraic equations, which is easily accomplished using computer algebra systems. As a result, applying the improved modified Tanh approach to the two-dimensional (2+1) KPSKR equation is not meant to compete with the bilinear method for discovering more exotic solutions like molecules or complicated hybrid patterns. Rather, it serves an important complementary role.

The manuscript is structured as below: The section “The mathematical approach” discusses the improved modified extended Tanh function method. The section “Exact solution procedures of (KPSKR) equation” presents the method’s application on the KPSKR equation and its outcomes. The section “Illustrations of the solutions” provides graphical representations of some solutions, and the section “Analysis of linear stability” concludes the manuscript.

## The mathematical approach

A powerful analytical technique for finding exact solutions to nonlinear partial differential equations (PDEs) is the improved modified extended Tanh function method. It represents an improvement over an extended Tanh method^28,29^. The improved modified extended Tanh function method introduces variations to enhance its effectiveness and provides accurate analytical solutions, which are essential for understanding the physical behavior of a system. It is capable of producing a variety of solution types, including solitary waves, periodic waves, and rational solutions, and simplifies the process of solving nonlinear and multidimensional equations.

The following is an explanation of the steps required in the improved modified extended Tanh function method^27^:

Assume having the following nonlinear partial differential equation (NLPDE):

2 $$\begin{aligned} G(z,z_t,z_x,z_{xx},z_{tx},....)=0. \end{aligned}$$

**Step 1**: Transform the PDE Eq. (2) into an ODE by introducing a traveling wave solution of the form

3 $$\begin{aligned} z(x,t)=z(\xi ), ~ \xi =c t+x,~ c\ne 0, \end{aligned}$$

wherein *c* stands for the traveling wave’s velocity. NLPDE in Eq. (2) then turns into:

4 $$\begin{aligned} F(z,z',z'',z''',....)=0, \end{aligned}$$

where $z'$ denotes the first derivative with respect to $\xi$, $z''$ denotes the second derivative with respect to $\xi$, $z^{n}$ denotes the $n^{th}$ derivative with respect to $\xi$.

**Step 2**: The following represents the solution to Eq. (4):

5 $$\begin{aligned} z(\xi )=\sum _{n=0}^{l}{a_n \phi ^n(\xi )}+\sum _{n=1}^{l}{b_{n} \phi ^{-n}(\xi )}, \end{aligned}$$

where the following differential equation (DE) is satisfied by $\phi (\xi )$:

6 $$\begin{aligned} \phi '(\xi )=\sqrt{d_0+d_1 \phi (\xi )+d_2 \phi ^2(\xi )+d_3 \phi ^3(\xi )+d_4 \phi ^4(\xi )}. \end{aligned}$$

**Step 3**: To find the value of *l* in Eq. (5), apply the balancing principle between the nonlinear term and the highest order linear term in Eq. (4).

**Step 4**: Substitute the assumed solution of Eq. (5) which satisfy Eq. (6) into the ODE equation (Eq. (4)).

**Step 5**: The parameters $a_n$, $b_n$ can be found by solving a series of equations that arises by setting all of the $\phi ^n$ coefficients of the resulting polynomial to zero using Mathematica software, Version 11.3.0.0. https://wolfram-mathematica.informer.com/11.2/.

**Step 6**: Different solutions can be raised by putting $d_0,d_1,d_2,d_3,d_4$ with distinct values as following:

**Case 1**: $$d_0=d_1=d_3=0$$

$$\begin{aligned} \phi (\xi )= & \sqrt{-\frac{d_2}{d_4}} \text {sech}\left( \sqrt{d_2} \xi \right) ,\quad d_2>0,~~d_4<0.\\ \phi (\xi )= & \sqrt{-\frac{d_2}{d_4}} \sec \left( \sqrt{-d_2} \xi \right) , \quad d_2<0,~~d_4>0. \end{aligned}$$

**Case 2**: $$d_1=d_3=0,d_0=\frac{d_2^2}{4d_4}$$

$$\begin{aligned} \phi (\xi )= & \sqrt{-\frac{d_2}{2 d_4}} \tanh \left( \sqrt{-\frac{d_2}{2}} \xi \right) ,\quad d_2<0,~~d_4>0.\\ \phi (\xi )= & \sqrt{\frac{d_2}{2 d_4}} \tan \left( \sqrt{\frac{d_2}{2}} \xi \right) ,\quad d_2>0,~~d_4>0.\\ \end{aligned}$$

**Case 3:** $$d_{3}=d_{4}=0$$

$$\begin{aligned} \phi (\xi )= & \sqrt{\dfrac{-d_{0}}{d_{2}}}\sin (\sqrt{-d_{2}}\xi ),\quad d_{0}>0,~~d_{1}=0,~~d_{2}<0.\\ \phi (\xi )= & \sqrt{\dfrac{d_{0}}{d_{2}}}\sinh (\sqrt{d_{2}}\xi ),\quad d_{0}>0,~~d_{1}=0,~~d_{2}>0.\\ \end{aligned}$$

**Case 4**: $$d_0=d_1=0$$, $$d_4>0$$

$$\begin{aligned} \phi (\xi )=\dfrac{1}{2}\sqrt{\dfrac{d_{2}}{d_{4}}}\left( 1+\tanh (\dfrac{1}{2} \sqrt{d_{2}}\xi )\right) ,\quad d_{2}>0,~~d_{3}=2\sqrt{d_{2}d_{4}}. \end{aligned}$$

**Step 7**: Inserting the previously determined values of $a_j,b_{-j}$ into Eq. (5) along with the previously general solutions of Eq. (6) yields several solutions for Eq. (2).

The following outcomes can be obtained by following the procedures described in the previous steps.

## Exact solution procedures of (KPSKR) equation

The steps of the improved modified extended Tanh method that were explained in the preceding section will be applied in this section to determine the exact solution for Eq. (1).

7 $$\begin{aligned} u(x,y,t)=z(\xi ), ~ \xi =x+y+c t. \end{aligned}$$

Substitute Eq. (7) into the original PDE Eq. (1) to obtain this ODE equation:

8 $$\begin{aligned}  &   (6+90 z) (z')^2+(c+\sigma +6z+45z^2) z''+15(z'')^{2}+30z'z^{(3)}+z^{(4)}+15zz^{(4)}+z^{(6)}=0. \end{aligned}$$

After applying the principle of balance between the highest-order linear term $z^{(6)}$ with nonlinear term $zz^{(4)}$ or $z'z'''$ in Eq. (8), we obtain $l=2$ using the equation $(l + 6 = 2 l + 4)$. Equation (8)’s solution can therefore be expressed as follows:

9 $$\begin{aligned} z(\xi )=a_0+a_1 \phi (\xi )+a_2 \phi ^2(\xi )+\dfrac{b_1}{\phi (\xi )}+\dfrac{b_2}{\phi ^2(\xi )}. \end{aligned}$$

**Case 1**: $$d_0=d_1=d_3=0$$

**Result (1)**

10 $$\begin{aligned} a_0= & \frac{1}{15} \left( -20 d_2-1\right) ,~d_4=-\frac{a_2}{4},~a_1=b_1=b_2=0,~d_2=\frac{\sqrt{-5 c-5 \sigma +1}}{4 \sqrt{5}}.\nonumber \\ u(x,y,t)= & \frac{1}{15} \left( -\sqrt{5} \sqrt{-5 c-5 \sigma +1}+3 \sqrt{5} \sqrt{-5 c-5 \sigma +1} ~\text {sech}^2\left( \frac{1}{2} \root 4 \of {-c-\sigma +\frac{1}{5}} (c t+x+y)\right) -1\right) ,\nonumber \\ & -5 c-5 \sigma +1>0. \end{aligned}$$

This is a representation of a bright soliton solution.

**Result (2)**

11 $$\begin{aligned} a_0= & \frac{1}{15} \left( -20 d_2-1\right) ,~d_4=-\frac{a_2}{4},~a_1=b_1=b_2=0,~d_2=-\frac{\sqrt{-5 c-5 \sigma +1}}{4 \sqrt{5}}.\nonumber \\ u(x,y,t)= & \frac{1}{15} \left( \sqrt{5} \sqrt{-5 c-5 \sigma +1}-3 \sqrt{5} \sqrt{-5 c-5 \sigma +1}~ \sec ^2\left( \frac{1}{2} \root 4 \of {-c-\sigma +\frac{1}{5}} (c t+x+y)\right) -1\right) ,\nonumber \\ & -5 c-5 \sigma +1>0. \end{aligned}$$

This is a representation of a singular periodic solution.

**Case 2**: $$d_1=d_3=0,~d_0=\frac{d_2^2}{4d_4}$$

**Result (1)**

12 $$\begin{aligned} a_0= & \frac{1}{15} \left( -20 d_2-1\right) ,~d_4=-\frac{a_2}{4},~a_1=b_1=b_2=0,~d_2=\frac{\sqrt{-5 c-5 \sigma +1}}{2 \sqrt{5}}.\nonumber \\ u(x,y,t)= & \frac{1}{15} \left( -2 \sqrt{5} \sqrt{-5 c-5 \sigma +1}-3 \sqrt{5} \sqrt{-5 c-5 \sigma +1} ~\tan ^2\left( \frac{1}{2} \root 4 \of {-c-\sigma +\frac{1}{5}} (c t+x+y)\right) -1\right) ,\nonumber \\ & -5 c-5 \sigma +1>0. \end{aligned}$$

This is a representation of a singular periodic solution.

**Result (2)**

13 $$\begin{aligned} a_0= & \frac{1}{15} \left( -20 d_2-1\right) ,~d_4=-\frac{a_2}{4},~a_1=b_1=b_2=0,~d_2=-\frac{\sqrt{-5 c-5 \sigma +1}}{2 \sqrt{5}}.\nonumber \\ u(x,y,t)= & \frac{1}{15} \left( 2 \sqrt{5} \sqrt{-5 c-5 \sigma +1}-3 \sqrt{5} \sqrt{-5 c-5 \sigma +1} ~\tanh ^2\left( \frac{1}{2} \root 4 \of {-c-\sigma +\frac{1}{5}} (c t+x+y)\right) -1\right) ,\nonumber \\ & -5 c-5 \sigma +1>0. \end{aligned}$$

This is a representation of a hyperbolic solution.

**Result (3)**

14 $$\begin{aligned} a_0= & \frac{1}{15} \left( -20 d_2-1\right) ,~a_1=b_1=a_2=0,~d_4=-\frac{d_2^2}{b_2},~d_2=\frac{\sqrt{-5 c-5 \sigma +1}}{2 \sqrt{5}}.\nonumber \\ u(x,y,t)= & \frac{1}{15} \left( -2 \sqrt{5} \sqrt{-5 c-5 \sigma +1}-3 \sqrt{5} \sqrt{-5 c-5 \sigma +1}~ \cot ^2\left( \frac{1}{2} \root 4 \of {-c-\sigma +\frac{1}{5}} (c t+x+y)\right) -1\right) ,\nonumber \\ & -5 c-5 \sigma +1>0. \end{aligned}$$

This is a representation of a singular periodic solution.

**Result (4)**

15 $$\begin{aligned} a_0= & \frac{1}{15} \left( -20 d_2-1\right) ,~a_1=b_1=a_2=0,~d_4=-\frac{d_2^2}{b_2},~d_2=-\frac{\sqrt{-5 c-5 \sigma +1}}{2 \sqrt{5}}.\nonumber \\ u(x,y,t)= & \frac{1}{15} \left( -2 \sqrt{5} \sqrt{-5 c-5 \sigma +1}-3 \sqrt{5} \sqrt{-5 c-5 \sigma +1}~ \coth ^2\left( \frac{1}{2} \root 4 \of {-c-\sigma +\frac{1}{5}} (c t+x+y)\right) -1\right) ,\nonumber \\ & -5 c-5 \sigma +1>0. \end{aligned}$$

This is a representation of a singular soliton solution.

**Result (5)**

16 $$\begin{aligned} a_0= & \frac{1}{15} \left( -20 d_2-1\right) ,~d_4=-\frac{a_2}{4},~b_2=-\frac{d_2^2}{d_4},~d_2=\frac{\sqrt{-5 c-5 \sigma +1}}{8 \sqrt{5}},~a_1=b_1=0.\nonumber \\ u(x,y,t)= & \frac{1}{60} \Bigg (-2 \sqrt{5} \sqrt{-5 c-5 \sigma +1}-3 \sqrt{5} \sqrt{-5 c-5 \sigma +1} ~\tan ^2\left( \frac{1}{4} \root 4 \of {-c-\sigma +\frac{1}{5}} (c t+x+y)\right) \nonumber \\ & -3 \sqrt{5} \sqrt{-5 c-5 \sigma +1}~ \cot ^2\left( \frac{1}{4} \root 4 \of {-c-\sigma +\frac{1}{5}} (c t+x+y)\right) -4\Bigg ),\quad \quad \quad -5 c-5 \sigma +1>0. \end{aligned}$$

This is a representation of a singular periodic solution.

**Result (6)**

17 $$\begin{aligned} a_0= & \frac{1}{15} \left( -20 d_2-1\right) ,~d_4=-\frac{a_2}{4},~b_2=-\frac{d_2^2}{d_4},~d_2=-\frac{\sqrt{-5 c-5 \sigma +1}}{8 \sqrt{5}},~a_1=b_1=0.\nonumber \\ u(x,y,t)= & \frac{1}{60} \Bigg (-2 \sqrt{5} \sqrt{-5 c-5 \sigma +1}-3 \sqrt{5} \sqrt{-5 c-5 \sigma +1} ~\tanh ^2\left( \frac{1}{4} \root 4 \of {-c-\sigma +\frac{1}{5}} (c t+x+y)\right) \nonumber \\ & -3 \sqrt{5} \sqrt{-5 c-5 \sigma +1} ~\coth ^2\left( \frac{1}{4} \root 4 \of {-c-\sigma +\frac{1}{5}} (c t+x+y)\right) -4\Bigg ),\quad \quad \quad -5 c-5 \sigma +1>0. \end{aligned}$$

This is a representation of a singular soliton solution.

**Case (3)**: $$d_1=d_3=d_4=0$$

**Result (1)**

18 $$\begin{aligned} a_0= & \frac{1}{15} \left( -20 d_2-1\right) ,~a_1=b_1=a_2=0,~d_0=-\frac{b_2}{4},~d_2=\frac{\sqrt{-5 c-5 \sigma +1}}{4 \sqrt{5}}.\nonumber \\ u(x,y,t)= & \frac{1}{15} \left( -\sqrt{5} \sqrt{-5 c-5 \sigma +1}-3 \sqrt{5} \sqrt{-5 c-5 \sigma +1} ~\text {csch}^2\left( \frac{1}{2} \root 4 \of {-c-\sigma +\frac{1}{5}} (c t+x+y)\right) -1\right) ,\nonumber \\ & -5 c-5 \sigma +1>0. \end{aligned}$$

This is a representation of a singular soliton solution.

**Result (2)**

19 $$\begin{aligned} a_0= & \frac{1}{15} \left( -20 d_2-1\right) ,~a_1=b_1=a_2=0,~d_0=-\frac{b_2}{4},~d_2=-\frac{\sqrt{-5 c-5 \sigma +1}}{4 \sqrt{5}}.\nonumber \\ u(x,y,t)= & \frac{1}{15} \left( -\sqrt{5} \sqrt{-5 c-5 \sigma +1}-3 \sqrt{5} \sqrt{-5 c-5 \sigma +1}~ \csc ^2\left( \frac{1}{2} \root 4 \of {-c-\sigma +\frac{1}{5}} (c t+x+y)\right) -1\right) ,\nonumber \\ & -5 c-5 \sigma +1>0. \end{aligned}$$

This is a representation of a singular periodic solution.

**Case (4)**: $$d_0=d_1=0$$, $$d_{4}=\dfrac{d_3^2}{4d_2}$$

**Result (1)**

20 $$\begin{aligned} a_0= & \frac{1}{15} \left( -5 d_2-1\right) ,~a_2=-\frac{d_3^2}{d_2},~d_3=-\frac{a_1}{2},~d_2=\frac{\sqrt{-5 c-5 \sigma +1}}{\sqrt{5}},~b_1=b_2=0.\nonumber \\ u(x,y,t)= & \frac{1}{15} \left( 2 \sqrt{5} \sqrt{-5 c-5 \sigma +1}-3 \sqrt{5} \sqrt{-5 c-5 \sigma +1}~\tanh ^2\left( \frac{1}{2} \root 4 \of {-c-\sigma +\frac{1}{5}} (c t+x+y)\right) -1\right) ,\nonumber \\ & -5 c-5 \sigma +1>0. \end{aligned}$$

This is a representation of a hyperbolic solution.

## Illustrations of the solutions

**Fig. 1:**
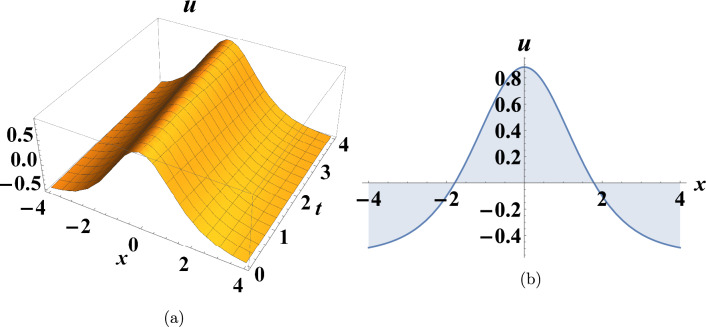
Graphical simulations of Eq. (10).

**Fig. 2:**
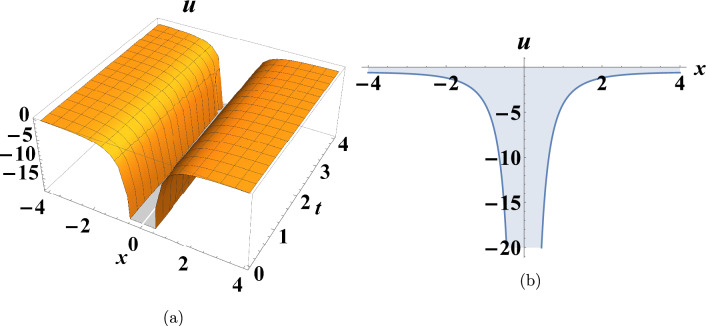
Graphical simulations of Eq. (18).

**Fig. 3:**
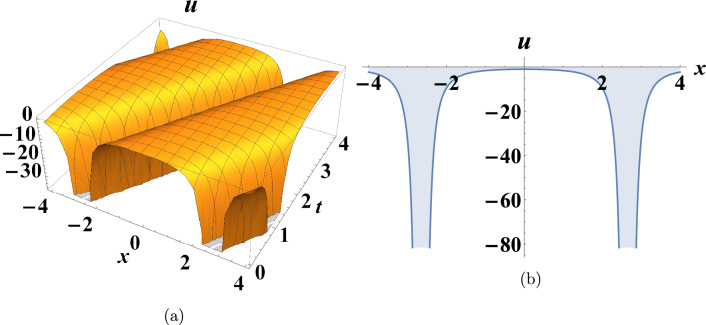
Graphical simulations of Eq. (11).

This section presents some graphs that illustrate the extent of change between the dependent variable u(x,t) and the independent variables (x and t) for some of the various results obtained in the previous section to clarify their characteristics. Figure 1 shows a depiction graphically of a bright soliton of Eq. (10) when $c=0.175,~y=0,~\sigma =-2$. Figure 2 shows a depiction graphically of a singular soliton of Eq. (18) when $c=0.06,~y=0,~\sigma =-2$. Figure 3 shows a depiction graphically of a singular periodic solution of Eq. (11) when $c=-1.225,~y=0,~\sigma =-0.545$.

## Analysis of linear stability

This section examines the linear stability properties of the (2+1) KPSKR equation. Linear stability analysis (LSA) is a fundamental mathematical tool used to determine the stability of steady-state solutions to a dynamical system, such as a differential equation.

If a small perturbation applied to an equilibrium state grows, indicating instability, or decays, indicating stability, or oscillates without changing amplitude, indicating neutral stability. The behavior of a very small perturbation is governed by a simplified linear version of the original nonlinear partial differential system. The eigenvalues, or dispersion relation, of this linear system determine the growth rate of these perturbations. In case the real component of any eigenvalue is positive, the perturbation grows exponentially over time, implying that the underlying solution is linearly unstable. In contrast, if all eigenvalues have negative real components, the perturbations decay, and the solution becomes stable. For entirely imaginary eigenvalues, the perturbations neither decrease nor increase, demonstrating neutral stability.

In this part, the analysis of linear stability of Eq. (1)^30^ will be examined applying the analysis of linear stability. The following gives the perturbed solution of the given equation:

21 $$\begin{aligned} u(x,y,t)=\lambda U(x,y,t)+q, {\quad 0 < \lambda \ll 1}, \end{aligned}$$

We assume the solution consists of the constant background plus a very small, spatially-dependent perturbation *U*(*x*, *y*, *t*). The steady state solution of Eq. (1) is denoted by *q*. When Eq. (21) is substituted into Eq. (1), the result is

22 $$\begin{aligned} & 90 \lambda U_x^2 (q+\lambda U)+45 U_{\text {xx}} (q+\lambda U)^2+6 U_{\text {xx}} (q+\lambda U)+15 U_{\text {xxxx}} (q+\lambda U)+6 \lambda U_x^2+30 \lambda U_x U_{\text {xxx}}+U_{\text {xt}}\nonumber \\ & \quad +15 \lambda U_{\text {xx}}^2+U_{\text {xxxx}}+U_{\text {xxxxxx}}+\sigma U_{\text {yy}}=0. \end{aligned}$$

By linearization Eq. (22), we get

23 $$\begin{aligned} & 45 q^2 U_{\text {xx}}+6 q U_{\text {xx}}+15 q U_{\text {xxxx}}+U_{\text {xt}}+U_{\text {xxxx}}+U_{\text {xxxxxx}}+\sigma U_{\text {yy}}=0. \end{aligned}$$

Assume that the solution to (23) takes the following form:

24 $$\begin{aligned} U(x,y,t)=\rho e^{i(M x+R y+t W)}, \end{aligned}$$

where the normalized wave numbers are *M* and *R*. By substituting (24) to (23), we can then solve for *W* and obtain

25 $$\begin{aligned} W=\frac{M^6+15 M^4 q+M^4-45 M^2 q^2-6 M^2 q-R^2 \sigma }{M}. \end{aligned}$$

**Fig. 4:**
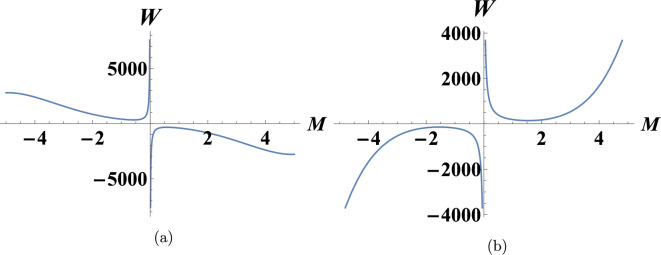
The relationship of dispersion between wave and frequency given that **a** $R=10,~q=-2.5,~\sigma =1$. **b** $R=10,~q=0.65,~\sigma =-2$.

The propagation relations for Eq. (25) are shown in Fig. 4. In a particular time frame, the sign of *W* show that the solution will either grow or decay. Every superposition of solutions in the kind of $e^{i(M x+R y+W t)}$ will decay at every value of *M*, and the steady state will be stable when the sign of *W* is negative. If *W* is positive for some *M* values, the steady state becomes unstable as some components of the superposition quickly increase over time. If the maximum *W*(*M*, *N*) is exactly 0, the dispersion is considered neutrally (marginally) stable and the system neither grows nor decays over time.

## Conclusion

This paper derives a new accurate solution to the nonlinear (2+1) KPSKR equation using the improved modified extended Tanh function technique. Regarding the provided model, several solutions have been obtained. Such solutions encompass hyperbolic and singular periodic solutions, as well as {bright and singular} solitons. Additionally, using Mathematica software, three-dimensional and two-dimensional charts are used to demonstrate how these solutions behave. The extracted solutions show the possibility of obtaining a stable wave for the proposed model. The unique balance between the nonlinear and dispersive effects in this model results in these stable propagating waves. These results will be helpful for the development of optical communication systems. This dependable and effective approach can be used to solve different models in physics and other fields of applied science.

## Data Availability

The datasets used and/or analyzed during the current study are available from the corresponding author upon reasonable request.
